# Community Participation in Native American and White Individuals After Traumatic Brain Injury: A 5‐Year Longitudinal Model Systems Study

**DOI:** 10.1002/brb3.70195

**Published:** 2024-12-22

**Authors:** Jack D. Watson, Paul B. Perrin, Bridget Xia, Juan Carlos Arango‐Lasprilla

**Affiliations:** ^1^ Department of Psychology Virginia Commonwealth University Richmond Virginia USA; ^2^ School of Data Science and Department of Psychology University of Virginia Charlottesville Virginia USA; ^3^ Central Virginia Veterans Affairs Health Care System Richmond Virginia USA

**Keywords:** community participation, disparities, Native American, rehabilitation, traumatic brain injury

## Abstract

**Purpose/Objective:**

This study examined (a) differences in demographic and injury‐related characteristics following traumatic brain injury (TBI) between Native American and White individuals; (b) differences in community participation between Native American and White individuals with TBI at 1, 2, and 5 years after TBI; and (c) whether demographic or injury‐related characteristics account for community participation disparities.

**Research Method/Design:**

A sample of 63 Native American individuals demographically matched to 63 White individuals (*n* = 126) was enrolled while on acute rehabilitation for moderate or severe TBI. Baseline demographic and injury‐related characteristics were collected at this time and the Participation Assessment with Recombined Tools (PART‐O) measure of community participation at 1, 2, and 5 years after TBI.

**Results:**

Native American individuals were less likely to have private insurance and be employed at the time of injury and more likely to have had lower educational attainment and engaged in binge drinking in the month prior to TBI compared to White individuals. Native American individuals demonstrated lower Out and About scores but statistically equivalent Social and Productivity scores. The difference in Out and About scores did not change as a function of time, and the overall difference between the two groups dissipated with the inclusion of statistically different sociodemographic variables.

**Conclusions/Implications:**

Clinicians can provide culturally sensitive, patient‐ and family‐centered care by conducting comprehensive interviews and identifying strengths as well as risk factors that enhance or hinder community participation after TBI in Native American individuals.

## Graphical abstract

This study examined longitudinal trajectories and disparities of community participation in Native American individuals over the 5 years following TBI. The findings indicated that Native American individuals experienced reduced community participation after injury compared to White individuals and that Native American individuals were less likely to have private insurance and be employed at baseline and more likely to have had lower educational attainment and engaged in binge drinking in the month prior to TBI.

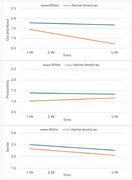

## 1 Introduction

Injury is the primary cause of preventable death in Native American individuals under age 45, who are at risk for elevated morbidity and mortality after traumatic brain injury (TBI; Peterson et al. 2019). As of the early 2000s, incidence for TBI requiring hospitalization for Native American individuals was 75/100,000 people (Langlois et al. 2003; Rutland‐Brown et al. 2005); however, from 2008 to 2014, TBI‐related hospitalizations for Native American individuals grew by 32%, and TBI‐related deaths increased by 13% (Peterson et al. 2019). Motor vehicle crashes were the chief cause of TBI in Native American individuals under the age of 55, with falls, intentional self‐harm, and assault representing other leading causes (Peterson et al. 2019). For Native American individuals, risk factors for experiencing TBI include living in a rural location, being male, substance use (Zeiler and Zeiler 2017), and violence (i.e., assault and intimate partner violence; Blackmer and Marshall 1999; Linton and Kim 2014; Linton 2015, Kim 2016). TBI leads to higher post‐injury hospitalization rates for Native American individuals than other individuals with TBI (Rutland‐Brown et al. 2005). Native American individuals living with TBI are far more likely to develop a mood or anxiety disorder (Nelson et al. 2007) and face challenges with healthcare access, employment, and functional independence (Fuentes et al. 2016; Watson et al. 2024; Whitfield and Lloyd 2008).

Community participation is a general construct within the disability literature that refers to overall functioning at the societal level post‐injury (Bogner et al. 2013; Takada et al. 2016). It can be conceptualized as the degree to which an individual is able to engage in a normal pattern of social and community life following rehabilitation (Lee, McCormick, and Austin 2001) and is accompanied by an increase in quality of life (Kuipers and Lancaster 2000). Community participation is one of the most important aspects of rehabilitation after TBI (Jacobsson, Westerberg, and Lexell. 2010; Kuipers and Lancaster 2000; Lee, McCormick, and Austin 2001). While conceptions of community participation may vary across diverse cultural and ethnic groups (Sander et al. 2011), there is a paucity of literature examining outcomes following TBI for Native American individuals and even fewer examining health disparities (Lakhani, Townsend, and Bishara 2017; Watson et al. 2024; Whitfield and Lloyd 2008; Zeiler and Zeiler 2017).

Experiencing a TBI makes individuals less likely to engage with their community, return to pre‐injury roles, and be able to live independently, as well as more likely to feel lonely and experience social interaction difficulties (Corrigan, Smith‐Knapp, and Granger 1997; Erler et al. 2018; Hoofien et al. 2001; Pierce and Hanks 2006). It is common for individuals with TBI to face increased likelihood for divorce, difficulty attaining and maintaining employment, and decreased involvement in leisure activities (Corrigan, Smith‐Knapp, and Granger 1997; Hoofien et al. 2001; Sander et al. 2011). Disparities in community participation following TBI have been documented for individuals from racial/ethnic minority groups (Arango‐Lasprilla et al. 2007; Arango‐Lasprilla et al. 2008; Hart et al. 2005; Gary, Arango‐Lasprilla, and Stevens 2009). Native American individuals may face similar difficulties following TBI (Lakhani, Townsend, and Bishara 2017; Zeiler and Zeiler 2017).

To date, no research has examined community participation within a Native American sample. Given the gap in the literature, it is critical to explore potential disparities in community participation within the Native American community after TBI. As a result, the purposes of the current study were to examine (a) differences in demographic and injury‐related characteristics following TBI between Native American and White individuals; (b) differences in community participation between Native American and White individuals with TBI at 1, 2, and 5 years after TBI; and (c) whether demographic or injury‐related characteristics account for some community participation disparities.

## 2 Method

### 2.1 Transparency and Openness

We report how we determined our sample size, all data exclusions, all manipulations, and all measures in the study, and we follow JARS (Kazak 2018). All data analysis syntax and outputs as well as research materials are available via request to the corresponding author. The data are publicly available through an external data request to the TBI Model System (TBIMS) National Data and Statistical Center (https://www.tbindsc.org/researchers.aspx). Data were analyzed using IBM SPSS Statistics version 29. The study's design and its analysis were not pre‐registered.

### 2.2 Procedure

The study received approval from the TBIMS National Data and Statistical Center to use the public TBIMS National Database. All TBIMS medical and follow‐up centers received relevant Institutional Review Board approval. Informed consent was obtained prior to study enrollment from all TBIMS participants or, when necessary, a legal proxy (e.g., spouse). All data were collected through medical records, patient interviews, direct examination of patients, data collection forms, or interviews with a legal proxy. Data were collected at baseline then 1, 2, 5, and every 5 years thereafter following discharge from inpatient rehabilitation. Follow‐up data collection was done by phone, in‐person, or by mail. For the current study, only data from the first 5 years following discharge were used.

### 2.3 Participants

To be eligible for enrollment in the TBIMS, individuals are required to meet the following criteria: (1) sustained a TBI which resulted in (a) Glasgow Coma Scale (GCS) score < 13, (b) posttraumatic amnesia (PTA) > 24 h, (c) loss of consciousness > 30 min, or (d) neuroimaging evidence of abnormality caused by intracranial trauma; (2) received medical care for TBI within 72 h of the injury; (3) enrolled and completed inpatient rehabilitation at an approved TBIMS site; and (4) was 16 years of age or older at the time of the injury.

The current study included all individuals who identified as solely Native American (i.e., did not select more than one race/ethnicity) in the TBIMS database, had complete data for sex, age, and injury severity (as measured by time spent in PTA), and had at least one complete data point for community participation at years 1, 2, or 5 post‐injury. Each Native American participant was demographically matched on sex, age, and PTA category to a White individual with TBI for comparison. Further, matched White participants needed to have at least one complete data point for community participation, with preference given to having the same time point of data. For example, when the Native American participant had data from year 2 but not from years 1 or 5, the White participant with the most similar time point of data was matched. If multiple White individuals fit these criteria, the one appearing first in the database was selected to ensure consistency. The order of participants in the database was randomized to account for possible selection bias based on the time of enrollment.

There were a total of 90 Native American identifying individuals in the original dataset; however, this number was reduced to 63, as participants were included for the proposed study only if they had at least one complete community participation data point (years 1, 2, or 5) and complete data for age, sex, and PTA. Demographic and injury‐related characteristics of the 126 demographically matched individuals can be seen in Table 1. Information on completeness of data can be found in Table 2. Missing data could occur for refusal to participate, not available at the time of data collection, incorrect contact information at follow‐up, not being physically or cognitively able to participate, discontinued use of the variable, and death.

**TABLE 1:** Characteristics of Individuals with TBI.

Variable	Native American	White	*p* value
Age at injury, *M* (SD)	37.84 (16.16)	37.84 (16.12)	*p =* 1.000
Sex, *n* (%)			*p =* 1.000
Male	40 (63.50%)	40 (63.50%)	
Female	23 (36.50%)	23 (36.50%)	
Years of education pre‐injury, *M* (SD)	12.00 (2.44)	13.26 (3.05)	*p =* 0.015
Employment at injury, *n* (%)			*p =* 0.002
Employed	31 (49.20%)	48 (76.20%)	
Not employed	32 (50.80%)	15 (23.80%)	
Annual earning, *n* (%)			*p =* 0.759
<9999	7 (19.40%)	8 (21.10%)	
10,000–19,999	8 (22.20%)	6 (15.80%)	
20,000–29,999	10 (27.80%)	8 (21.10%)	
30,000–39,999	4 (11.10%)	5 (13.20%)	
40,000–49,999	2 (5.60%)	2 (5.30%)	
50,000–59,999	1 (2.80%)	3 (7.90%)	
60,000–69,999	—	2 (5.30%)	
70,000–79,999	1 (2.80%)	—	
80,00– 89,999	1 (2.80%)	—	
90,000–99,999	1 (2.80%)	1 (2.60%)	
> 100,000	1 (2.80%)	3 (7.90%)	
Type of work, *n* (%)			*p =* 0.302
Blue collar	25 (80.60%)	33 (70.20%)	
White collar	6 (19.40%)	14 (29.80%)	
Cause of injury, *n* (%)			*p =* 0.187
Non‐violent	56 (88.90%)	60 (95.20%)	
Violent	7 (11.10%)	3 (4.80%)	
Insurance type, *n* (%)			*p =* 0.047
Private	23 (37.10%)	34 (54.80%)	
Non‐private	39 (62.90%)	28 (45.20%)	
Marital status, *n* (%)			*p =* 0.177
Married	40 (63.50%)	47 (74.60%)	
Not married	23 (36.50%)	16 (25.40%)	
Language spoken at home, *n* (%)			*p =* 0.291
English	50 (94.30%)	54 (98.20%)	
Other than English	3 (5.70%)	1 (1.80%)	
Illicit/non‐prescription drug use, n (%)			*p =* 0.056
Reported problematic use	35 (57.40%)	24 (40.00%)	
Did not report problematic use	26 (42.60%)	36 (60.00%)	
Cigarette use, *n* (%)			*p =* 0.966
Smoked prior to injury	9 (42.90%)	3 (37.50%)	
Did not smoke prior to injury	12 (57.10%)	5 (62.50%)	
Alcohol use, *n* (%)			*p =* 0.040
Reported problematic use	28 (44.40%)	13 (20.70%)	
Did not report problematic use	32 (50.80%)	45 (71.40%)	
Days spent in PTA, *M* (SD)	25.70 (27.33)	21.94 (13.92)	*p =* 0.367

**TABLE 2:** Data missingness.

	Native American		White	
Variable	# With data	Missing (%)	# With data	Missing (%)
One‐Year Out and About	50	20.63	46	26.98
Two‐Year Out and About	48	23.81	49	22.22
Five‐Year Out and About	33	47.62	49	22.22
				
One‐Year Productivity	50	20.63	46	26.98
Two‐Year Productivity	49	22.22	50	20.63
Five‐Year Productivity	33	47.62	49	22.22
				
One‐Year Social	50	20.63	46	26.98
Two‐Year Social	48	23.81	50	20.63
Five‐Year Social	33	47.62	49	22.22

### 2.4 Measures

#### 2.4.1 Demographic and Injury‐Related Variables

The TBIMS study assesses a large number of demographic, injury‐related, and outcome variables. The following demographic and injury‐related characteristics were examined in the current study: marital status, years of education, annual earnings, employment status at the time of injury, type of employment (blue vs. white collar), violence as a cause of injury, language spoken at home, insurance type (private vs. other), and problematic alcohol use (binge drinking in the month prior to experiencing a TBI). All of these variables were examined through single‐item assessment or an electronic health records system. These items are typically used within rehabilitation research as either predictors of or explanations for health disparities (Arango‐Lasprilla, Watson, Ertl, et al. 2023; Arango‐Lasprilla, Watson, Merced, et al. 2023; Arango‐Lasprilla et al. 2022; Erler et al. 2018).

#### 2.4.2 Community Participation

Participation Assessment with Recombined Tools‐Objective (PART‐O). The PART‐O (Bogner et al. 2013) is a 17‐item measure developed to examine long‐term community participation and social function. It measures three domains of community participation: Productivity (e.g., hours spent working or in education), Out and About (e.g., how frequently the participant leaves their home to engage with their community), and Social Relations (e.g., quality of friendships or frequency of visitation with friends/family). To score the PART‐O, each domain subscale is averaged, producing a score of 0–5, and the subscale scores are then summed for a total score. For the proposed study, all three individual subscales were used; however, the overall score w not.

### 2.5 Data Analysis

#### 2.5.1 Descriptive, Assumption‐Based, and Missingness Analyses

All analyses were conducted using IBM SPSS Statistics version 29. Descriptive statistics were calculated and examined for the study sample (Table 1). The following demographic variables were examined: age, sex, annual earnings, language spoken at home, years of education, marital status, employment status, type of employment (blue vs. white collar), type of insurance (private vs. other), and problematic alcohol use. Additionally, descriptive statistics also reported the injury‐related variables of injury severity (as measured by time spent in PTA) and whether violence was the cause of injury. Analysis of variance (ANOVA) tests and 𝜒^2^ analyses were used where appropriate to examine the demographic and injury‐related characteristics for significant differences between the two groups.

Normality tests were performed on the outcome variable (community participation as measured by the PART‐O) to assess the shape of the distribution and transform if necessary. The percentage of missing data at 1, 2, and 5 years post‐discharge was reported (Table 2), and Little's Missing Completely at Random (MCAR) was used to assess the degree these data were missing at random. Full information maximum likelihood (FIML) estimation was used to include participants with missing data.

#### 2.5.2 Primary Analyses

An unconditional growth model was conducted with only the intercept, time, and time × time as fixed effects predictors to determine the best curvature model (e.g., straight or quadratic). A ‐2 log likelihood (‐2LL) for each successive model with a critical 𝜒^2^ value of significant difference at = 0.05 and > 3.841 drop from the previous model (at one degree of freedom) was used to determine adequate model curvature. Time was recoded as 0 (1 year), 1 (2 years), and 4 (5 years).

##### 2.5.2.1 Primary Set 1

The next set of hierarchical linear models (HLMs) assessed whether there was a difference in the three community participation subscales over time between the Native American and White groups. A follow‐up HLM for each subscale incorporated an interaction term between time and race/ethnicity to determine if the difference in community participation outcomes occurred differentially as a function of time.

##### 2.5.2.2 Primary Set 2

For the second primary set, the same analyses were conducted with the addition of the demographic and injury‐related covariates that differed significantly between the two groups which were centered or given a reference of zero. This HLM determined the degree to which these covariates accounted for some of the disparities in community participation. Potential covariates included marital status, years of education, annual earnings, employment status at the time of injury, type of employment (blue vs. white collar), violence as a cause of injury, language spoken at home, type of insurance, and substance use (alcohol, drugs, and tobacco); however, only years of education, employment at the time of injury, type of insurance, and alcohol use were significantly different between the two groups (Table 1). Therefore, this final set of HLMs only used those four covariates.

## 3 Results

### 3.1 Descriptive and Normality Analyses

**FIGURE 1:**
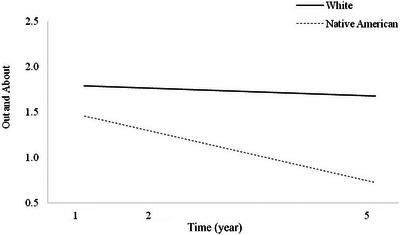
Main effect of race on Out and About trajectories.

Means (*M*s) and standard deviations (SDs) for all three PART‐O subscales (Out and About, Productivity, and Social) separated by race are shown in Table 3. Normality tests on all three subscales demonstrated they were normally distributed. Correlation coefficients between all potential covariates demonstrated no problematic multicollinearity (all *r* < 0.70). Further, of the potential covariates (Table 1; Figure 1) assessed via ANOVA or 𝜒^2^ tests, only years of education (*p* = 0.015), employment at the time of injury (*p* = 0.002), type of insurance held (*p* = 0.047), and alcohol use (*p* = 0.040) were significantly different between Native American and White individuals with TBI at baseline (Table 1).

**TABLE 3:** PART‐O subscale means and standard deviations by race.

Variable	Native American	White
One‐Year Out and About, *M* (SD)	1.314 (0.678)	1.738 (0.826)
Two‐Year Out and About, *M* (SD)	1.460 (0.746)	1.832 (0.765)
Five‐Year Out and About, *M* (SD)	1.434 (0.762)	1.663 (0.889)
		
One‐Year Productivity, *M* (SD)	1.047 (0.844)	1.341 (0.944)
Two‐Year Productivity, *M* (SD)	1.034 (0.782)	1.447 (0.961)
Five‐Year Productivity, *M* (SD)	1.162 (0.993)	1.327 (1.060)
		
One‐Year Social, *M* (SD)	2.263 (0.953	2.437 (0.823)
Two‐Year Social, *M* (SD)	2.340 (0.934)	2.526 (0.792)
Five‐Year Social, *M* (SD)	2.033 (1.194)	2.243 (1.015)

### 3.2 Curvature Analysis and Missing at Random

Results from Little's MCAR suggested that data for all three subscales were missing completely at random (*χ* ^2^ [3] = 4.99, *p* = 0.173). For the missing data, HLM's FIML was used to retain all participants despite missingness. An unconditional growth model was conducted with only the intercept and time as fixed effect predictors and then a second model with the addition of time × time to determine the best curvature model (e.g., straight or quadratic) for the HLMs. A ‐2 log likelihood (‐2LL) for each successive model with a critical 𝜒^2^ value of significant difference at *α* = 0.05 and > 3.841 drop from the previous model (at one degree of freedom) was used to determine the best fitting model curvature for each subscale over time. The curvature comparisons indicated that a straight line best fit all three subscales of the PART‐O.

### 3.3 Primary Analyses

#### 3.3.1 PART‐O Out and about

##### 3.3.1.1 Primary Set 1

There was a significant main effect of race on Out and About trajectories (*p* = 0.006), indicating that Native American individuals had worse Out and About trajectories over time than White individuals with TBI (Figure 1). There was not, however, a significant interaction effect for time × race (Table 4; *p* = 0.355), indicating that the slopes of these Out and About trajectories did not differ significantly by race (Figure 1).

**TABLE 4:** Predictors of the PART‐O subscales.

	Out and About		Productivity		Social	
Predictor	*b*weight	*p* value	*b*weight	*p* value	*b*‐weight	*p* value
Set 1: Race						
Intercept	1.753	<0.001	1.329	<0.001	2.478	<0.001
Time	−0.022	0.274	0.015	0.567	−0.050	0.049
Native American vs. White	−0.339	0.006	−0.259	0.077	−0.202	0.180
Set 1: Race interaction with time						
Intercept	1.780	<0.001	1.378	<0.001	2.494	<0.001
Time	−0.037	0.152	−0.013	0.697	−0.059	0.074
Native American vs. White	−0.398	0.004	−0.362	0.030	−0.2374	0.164
Time × Race	0.037	0.355	0.067	0.198	0.022	0.660
Set 2: Race with covariates						
Intercept	0.832	0.005	—	—	—	—
Time	−0.012	0.551	—	—	—	—
Native American vs. White	−0.108	0.390	—	—	—	—
Education	0.053	0.015	—	—	—	—
Employment at Injury	0.067	0.612	—	—	—	—
Type of insurance	0.323	0.009	—	—	—	—
Alcohol use	−0.001	0.890	—	—	—	—

##### 3.3.1.2 Primary Set 2

Given that Out and About trajectories differed significantly as a function of race, a follow‐up HLM was run to attempt to account for this difference through sociodemographic and injury‐related characteristics that differed significantly between the two groups (Table 1). Years of education, employment at the time of injury, type of insurance, and alcohol use patterns were entered as covariates to the HLM (Table 4). Results indicated that these covariates accounted for the statistically different Out and About trajectories between the two racial groups, reducing the significance of race as a predictor (*p* = 0.390). Both education (*p* = 0.015) and type of insurance (private vs. other; *p* = 0.009) were significant predictors of Out and About trajectories (Table 4), likely accounting for the predictive power of race. Because the Out and About trajectory slopes did not differ significantly by race (i.e., the time × race effect was not significant), no respective follow‐up model including the covariates was run.

#### 3.3.2 PART‐O Productivity

##### 3.3.2.1 Primary Set 1

**FIGURE 2:**
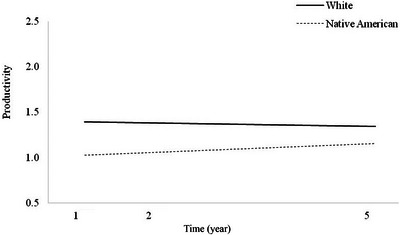
Main effect of race on Productivity trajectories.

There was no significant main effect of race on Productivity trajectories (Table 4; *p* = 0.077), though it was marginally significant. This suggests that Native American and White individuals with TBI have statistically equivalent Productivity scores over time, though the effect was in the anticipated direction of higher Productivity scores for the White group (Figure 2). Additionally, there was no significant interaction effect for time × race, again suggesting that Productivity scores did not diverge significantly over time as a function of race. Because neither model was significant, no follow‐up models with the statistically different covariates were run.

#### 3.3.3 PART‐O Social

##### 3.3.3.1 Primary Set 1

**FIGURE 3:**
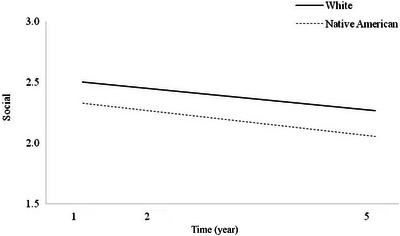
Main effect of race on Social trajectories.

There was no significant main effect of race on Social trajectories (Table 4; *p* = 0.180). This suggests that Native American and White individuals with TBI had statistically equivalent Social scores over time (Figure 3). Again, there was no significant interaction effect for time × race, indicating that Social scores did not diverge significantly over time as a function of race. Because neither model was significant, no follow‐up models with the statistically different covariates were run.

## 4 Discussion

The purpose of this study was to examine (a) differences in demographic and injury‐related characteristics following TBI between Native American and White individuals; (b) differences in community participation between Native American and White individuals with TBI at 1, 2, and 5 years after TBI; and (c) whether demographic or injury‐related characteristics account for some community participation disparities. Native American individuals were less likely to have private insurance and be employed at the time of injury and were more likely to have lower educational attainment and engage in binge drinking in the month prior to experiencing a TBI. Over the 5 years following TBI, Native American individuals demonstrated lower Out and About scores compared to White individuals; this difference remained relatively stable over time. Social and Productivity scores were statistically equivalent over time. The difference in Out and About scores did not change as a function of time, and the overall difference between the two groups dissipated with the inclusion of the statistically different sociodemographic variables.

The finding that Native American individuals with TBI were less likely to have private insurance is important in the context of health disparities experienced by this population (Clements et al. 2020; Cromer, Wofford, and Wyant 2019; Liddell 2020; Marrone 2007; Watson et al. 2024; Whitfield and Lloyd 2008). Type of insurance is a key predictor of worse cognitive and motor function in Native American individuals over the 5 years post‐TBI (Watson et al. 2024). Lack of private insurance may be associated with Native American individuals’ concern over the cost and reliability of healthcare services, suspicion of western medicine practices, and general provider distrust (Liddell 2020; Marrone 2007). Similar to large‐scale investigations of the sociodemographic characteristics of Native American individuals (Sarche and Spicer 2008), we found that Native American individuals with TBI were less likely to be employed at the time of TBI and had lower educational attainment compared to White individuals. Employment status may be linked to insurance status, as one's place of employment is often a primary means of acquiring health insurance.

Both educational attainment and type of insurance remained significant predictors of Out and About trajectories in the model including the covariates that differed significantly between the two groups. Thus, it is possible these either account for the racial differences in Out and About trajectories or are indicators of other unmeasured sources of variance (e.g., lack of access to healthcare). Given that higher educational attainment is a predictor of better functional recovery after TBI (Schneider et al. 2014), lower levels of education may predispose Native American individuals to worse cognitive outcomes following TBI (Watson et al. 2024). While the rates of alcohol use within the Native American community are variable (Cunningham, Solomon, and Muramoto 2016), substance use, especially alcohol, appears to be a risk factor for TBI in this population, with rates twice that of other racial/ethnic minorities (Linton, Jung Kim, and Kim 2016). Alcohol misuse may lead to adverse health and rehabilitation outcomes, impacting engagement in community participatory activities after TBI.

Despite the demographic and community participation disparities found in this study among Native American individuals with TBI, it is extremely important for rehabilitation science to also be aware of the unique strengths of this community that might be bootstrapped in future rehabilitation efforts after TBI. While there is no culturally informed measure of community participation for Native American individuals, there is a measure of spirituality, the Native American Spirituality Scale (NASS; Greenfield et al. 2015). The NASS was developed in conjunction with members of a Native American tribe to help capture aspects of spirituality that are contextually relevant to Native American individuals (Greenfield et al. 2015). Spirituality is important to many Native American individuals and is shown through Native American burial rituals and the Dakota Access pipeline protests (e.g., dancing, sitting in prayer circles). Greenfield et al. (2015) found that spirituality, especially spiritual behaviors, was strongly associated with both tribal identity and lower substance use. This finding indicates that spirituality may be an important cultural strength to leverage in rehabilitation and may be a buffer against negative outcomes for Native American individuals with TBI.

While, here is a paucity of research on Native American cultural/ethnic identity (Jones and Galliher 2015) existing research has suggested that many Native American individuals are proud of their cultural/ethnic identity (Jones and Galliher 2015). Given the close ties of spirituality and cultural/ethnic identity, it is possible that both aspects may play a role in defining community for Native American individuals and have important implications for clinical practice and future rehabilitation research. Racial/ethnic identity has been found to be a protective factor against a host of negative outcomes (Butler‐Barnes et al. 2018; Neblett et al. 2012). Similar results have been found within the disability community, suggesting that strong disability identity and/or pride may protect against negative experiences (e.g., discrimination) and outcomes (e.g., lower self‐esteem; Bogart et al. 2018). Many studies in Native American health disparities research are primarily deficit‐focused and decontextualized; however, understanding resilience and protective factors is important for reducing risk for adverse health outcomes and promoting healthy behavior in Native American communities (Oré et al. 2016). Connectedness to community, communal mastery, and engagement in cultural and spiritual practices are all important aspects of cultural resilience within Native American communities (John‐Henderson, White, and Crowder 2023). Therefore, it is important to account for the impact of historical context and sociocultural and geopolitical factors on Native American communities, and it is equally as vital for health disparities researchers to conduct culturally nuanced and informed research.

### 4.1 Limitations and Future Directions

The current study has several limitations that present opportunities for future research. The PART‐O may not accurately map onto Native American conceptualizations of community; by measuring the frequency of engaging in community activities, it does not capture cultural factors that can affect the meaningfulness or patterns of participatory activities in Native American individuals with TBI. Future research may wish to develop culturally responsive measures of community participation to capture long‐term outcomes post‐TBI.

Native American individuals with TBI are uniquely situated within the United States in that they have ties with multiple forms of identity that may act as either a buffer against negative outcomes or a generator of positive outcomes. Native American individuals with TBI may have multiple cultural/ethnic identities (e.g., Native American or Native American and some other race/ethnicity), tribal affiliation, and disability identity. Tribal affiliation is a unique identity category for individuals from the Native American communities that may offer additional benefits beyond Native American identity. Future research should explore how tribal identity in addition to Native American identity or the intersection of Native American, tribal, and disability identity may affect rehabilitation outcomes.

The Native American community has a plethora of unique characteristics, such as spirituality, strong sense of kinship that transcends tribal or geographic ties, stalwart leadership, and racial/ethnic and tribal identity. Such positive characteristics were not assessed in the present study due to lack of inclusion of these constructs within the TBIMS database. Future research may take a strengths‐based approach to uncover culturally and contextually specific characteristics of Native American individuals, which may have important clinical implications. Such research may be useful in creating culturally sensitive rehabilitation programs that lean on the strengths of the Native American community to increase the quality of care and maximize rehabilitation outcomes.

Access to healthcare resources, acculturation status, systemic biases, and comorbid conditions (e.g., diabetes) may have a significant impact on outcomes for Native American individuals with TBI (Marrone 2007; Whitfield and Lloyd 2008). We did not collect data on these important variables, which future research may wish to consider. The Indian Health Service (IHS) is a U.S. Department of Health and Human Services agency that provides healthcare services for Native American individuals who belong to federally recognized tribes. Specialty care, such as neurology, has been promoted and successfully sustained within the IHS, given the high prevalence of TBI in Native American individuals (Parko et al. 2024). Further research may consider examining factors affecting access to and use of IHS after TBI and ways to maximize quality of care, access, and utilization. Additionally, a larger sample size may help uncover more nuanced results and allow the examination of a larger number of possible covariates (e.g., comorbidities, distance to the nearest healthcare facility). Finally, the exact mechanism through which education, employment at the time of injury, type of insurance, and binge drinking in the month prior to experiencing a TBI might impact differences in community participation trajectories between Native American and White individuals is unclear. The nature of HLM does not allow for causal inference, and the aforementioned variables may be indicators of other unmeasured sources of variance (e.g., educational attainment may be an indicator of socioeconomic status; insurance type may affect the quality of healthcare). While the current study highlighted the predictive utility and relation among these variables and Out and About disparities over the 5 years post‐injury, future research may wish to examine possible causal pathways to better inform rehabilitation care and early intervention.

## 5 Conclusion

The current study was the first to examine longitudinal trajectories and disparities of community participation for Native American individuals with TBI when compared to a demographically matched sample of White individuals. The findings emphasize the important need for early assessment and intervention across key sociodemographic predictors and highlight gaps in the literature, particularly the lack of culturally informed measures of community participation and a dearth of research on culturally and contextually specific strengths that may be targeted to improve health outcomes. Clinicians are encouraged to provide culturally sensitive, patient‐ and family‐centered care by conducting comprehensive interviews and identifying strengths as well as risk factors that may enhance or hinder community participation when working with this underserved population. Given that Native American individuals are significantly less likely to engage with the healthcare system and have appropriate resources provided to them post‐discharge (Marrone 2007; Whitfield and Lloyd 2008), clinicians may consider targeting modifiable resources and advocating appropriately for these patients. Future research should develop culturally responsive measures of community participation to inform evidence‐based interventions to mitigate these health disparities and promote rehabilitation efforts.

## Author Contributions

**Jack D. Watson**: conceptualization, investigation, writing—review and editing, writing—original draft, methodology, visualization, formal analysis, project administration. **Paul B. Perrin**: conceptualization, investigation, writing—review and editing, writing—original draft, visualization, methodology, formal analysis, project administration, supervision. **Bridget Xia**: writing—review and editing, methodology, project administration. **Juan Carlos Arango‐Lasprilla**: conceptualization, supervision, writing—review and editing.

### Peer Review

The peer review history for this article is available at https://publons.com/publon/10.1002/brb3.70195

## Data Availability

The data that support the findings of this study are openly available in TBI Model Systems at https://www.tbindsc.org/StaticFiles/Documents/Using_TBIMS_NatDatabase_2020.pdf.
